# Stellungnahme zur Hospitalisierung von Kindern anlässlich von HNO-Eingriffen in Deutschland: Positionspapier der Arbeitsgemeinschaft Pädiatrische HNO-Heilkunde der Deutschen Gesellschaft für Hals-Nasen-Ohren-Heilkunde, Kopf- und Hals-Chirurgie

**DOI:** 10.1007/s00106-022-01215-4

**Published:** 2022-09-09

**Authors:** Jochen P. Windfuhr, Christian Sittel, Thomas Deitmer, Markus Jungehülsing, Thomas Grundmann, Rudolf Hagen, Ingo Baumann, Stephan Hackenberg, Arbeitsgemeinschaft Pädiatrische HNO-Heilkunde (AG PädHNO) der Deutschen Gesellschaft für Hals-Nasen-Ohren-Heilkunde, Kopf- und Hals-Chirurgie e. V.

**Affiliations:** 1 Kliniken Maria Hilf Mönchengladbach, Mönchengladbach, Deutschland; 2 Katharinenhospital, Stuttgart, Deutschland; 3 Deutsche Gesellschaft für Hals-Nasen-Ohren-Heilkunde, Kopf- und Hals-Chirurgie, Bonn, Deutschland; 4 Klinikum Ernst von Bergmann, Potsdam, Deutschland; 5 Asklepios Klinik Altona, Hamburg, Deutschland; 6 Universitätsklinikum, Würzburg, Deutschland; 7 Universitätsklinikum, Heidelberg, Deutschland; 8 Klinik für Hals-Nasen-Ohren-Heilkunde, Kopf- und Hals-Chirurgie, Universitätsklinikum RWTH Aachen, Pauwelsstr. 30, 52074 Aachen, Deutschland

**Keywords:** Pädiatrische HNO, Stationäre Überwachung, Nationale Umfrage, Pediatric ENT, Inpatient surveillance, National survey

## Abstract

Die stationäre Betreuung von Kindern nach Operationen im HNO-Bereich ist deutschlandweit heterogen organisiert. In dieser Stellungnahme kommentiert die Arbeitsgemeinschaft Pädiatrische HNO-Heilkunde eine nationale Umfrage zum Status quo der Kinderversorgung nach HNO-Eingriffen in deutschen Kliniken. Diese dient als Grundlage für die weitere interdisziplinäre Diskussion.

## Abstract (en)

The inpatient care of children after ENT surgery is organized heterogeneously across Germany. In this statement, the working group on Pediatric ENT comments a national survey on the status quo of child care after ENT interventions in German hospitals. The statement should serve as a basis for further interdisciplinary discussion.

## Einführung

Die stationäre Unterbringung von Kindern nach HNO-Eingriffen unterscheidet sich deutschlandweit teils erheblich. Das Spektrum reicht von der Betreuung auf HNO-ärztlich oder pädiatrisch geführten Belegabteilungen, HNO-Kliniken mit eigenen Kinderstationen, interdisziplinär geführten Dependancen von HNO-Kliniken in separaten Kinderkrankenhäusern bis zur Betreuung von HNO-Kindern auf (chirurgischen) Stationen einer Kinderklinik bzw. eines Kinderzentrums. Eine Betreuung von Kindern auf Stationen, die von der jeweiligen HNO-Abteilung räumlich getrennt sind, bedarf einer sehr sorgfältigen organisatorischen Strukturierung, um jedwede Risiken für die operierten Kinder zu minimieren. Im Fokus steht hier insbesondere die zeitkritische Beherrschung von Notfallsituationen, wie beispielsweise Blutungskomplikationen nach einer Tonsillektomie. Dies ist immer wieder Gegenstand bei den Diskussionen mit den Krankhausträgern und den interdisziplinären Kooperationspartnern. Die Arbeitsgemeinschaft Pädiatrische HNO-Heilkunde der Deutschen Gesellschaft für Hals-Nasen-Ohren-Heilkunde, Kopf- und Hals-Chirurgie führte 2019 eine deutschlandweite Umfrage zur Erfassung der postoperativen Versorgung von Kindern nach HNO-ärztlichen Eingriffen durch, deren Ergebnisse bereits publiziert wurden [1]. Die folgende Stellungnahme kommentiert die Ergebnisse der Umfrage kritisch und soll Grundlage sein für die weitere Diskussion innerhalb unserer Fachgesellschaft und mit den Krankenhaus- und Kostenträgern bzw. den beteiligten Partnerdisziplinen.

## Stellungnahme

Das Zahlenmaterial des statistischen Bundesamts belegt einen deutlichen Wandel der Krankenhauslandschaft in Deutschland (Tab. 1), von der auch die stationäre Versorgung von Krankheitsbildern der HNO-Heilkunde betroffen ist (Tab. 2; [2, 3]). Eine Reduzierung der Bettenverteilung und ein Rückgang der stationären Fälle ist ein klar ersichtlicher Trend, auch in der HNO-Heilkunde. Diese Entwicklung erfordert die Integration von Praxen sowie die Etablierung von Tageskliniken und interdisziplinären Zentren v. a. im Umfeld von Universitätskliniken und spezialisierten Maximalversorgern, um weiterhin eine hohe Qualität der Patientenbehandlung gewährleisten zu können [3]. Die Ausgestaltung von patientenorientierten Versorgungskonzepten mit einem professionellen Umfeld kann aber erheblich durch berufspolitische Vorgaben oder ökonomische Optimierungsgedanken gestört werden [4]. In Bezug auf die Versorgung pädiatrischer Krankheitsbilder unseres Fachgebiets kann dies im Einzelfall bedeuten, dass ein Krankenhausträger operierte Kinder prinzipiell in der pädiatrischen Abteilung unter Behandlungsführung der Pädiatrie und nicht mehr in der HNO-Klinik unterbringen will.

**Table 1:** 

	Öffentlich	Freigemeinnützig	Privat	Summe
2019	235.767	162.958	95.601	494.326
2018	238.907	164.081	95.204	498.192
2017	238.748	165.245	93.189	497.182
2016	238.803	166.858	93.057	498.718
2015	240.653	167.566	91.132	499.351
2014	240.195	169.477	91.008	500.680
2013	240.632	170.086	89.953	500.671
2012	240.180	171.276	90.019	501.475
2011	242.769	172.219	87.041	502.029
2010	244.254	173.457	85.038	502.749
2009	244.918	174.711	83.712	503.341
2008	246.423	177.085	79.852	503.360
2007	250.345	177.632	78.977	506.954
2006	260.993	180.200	69.574	510.767
2005	273.721	184.752	65.351	523.824
2004	280.717	189.334	61.282	531.333
2003	290.625	197.343	53.933	541.901

**Table 2:** 

Jahr	Fachabteilungen	Bettenzahl	Betten/100.000	Intensivbetten	Belegbetten	Fallzahl	Belegungstage
2017	635	9418	11,4	178	2467	571.660	2.045.309
2016	654	9821	11,9	165	2692	582.779	2.130.738
2015	665	10.019	12,3	153	2850	588.362	2.213.298
2014	686	10.263	12,7	156	3021	592.794	2.289.807
2013	690	10.456	13,0	153	3176	591.881	2.344.235
2012	702	10.686	13,3	149	3292	600.545	2.443.111
2011	714	10.878	13,6	138	3446	603.964	2.514.566
2010	730	11.128	13,6	135	3663	600.630	2.574.031
2009	739	11.313	13,8	146	3823	596.349	2.643.254
2008	745	11.608	14,1	145	4016	588.573	2.688.420
2007	755	11.784	14,3	145	4218	588.165	2.750.491
2006	763	12.070	14,7	153	4397	586.975	2.818.562
2005	793	12.522	15,2	162	4793	597.174	2.931.389
2004	820	13.100	15,9	150	5188	639.787	3.153.298
2003	844	13.600	16,5	170	5498	694.206	3.439.940
2002	854	13.819	16,8	163	5586	722.352	3.668.936
2001	864	14.025	17,0	160	5734	747.920	3.804.802
2000	876	14.324	17,4	154	5869	763.687	3.948.643
1999	881	14.509	17,7	155	5969	783.413	4.062.994
1998	895	14.711	17,9	153	6155	796.046	4.137.142
1997	901	15.073	18,4	158	6413	782.941	4.135.957
1996	915	15.398	18,8	144	6736	798.897	4.217.607
1995	924	15.760	19,3	139	7049	811.383	4.331.163
1994	934	16.212	19,9	154	7377	813.608	4.385.873

Im Gegensatz zu anderen Ländern gibt es in Deutschland nur wenige pädiatrisch-HNO-ärztliche Fachabteilungen und kein eigenständiges Fachgebiet oder eine Zusatzweiterbildung für „Pädiatrische HNO-Heilkunde“, insofern sind derzeit auch keine speziellen Versorgungsaufträge der jeweiligen Landesregierungen an die einzelnen Krankenhäuser zu erwarten [5]. Die postoperative Versorgung von pädiatrischen Krankenhausfällen unseres Fachgebiets lässt sich anhand einer aktuellen Umfrage unter 166 HNO-Hauptabteilungen und Universitätskliniken gut einschätzen. Inhaltlich ging es um die zeitkritische Versorgung von Blutungskomplikationen nach Tonsillektomie, da diese die Präsenz von operativ-fachärztlicher wie auch pflegerischer und nichtärztlicher, organisatorischer Kompetenz erfordert. Eine einheitliche Versorgungsform für die postoperative Betreuung von Kindern ließ sich bei der Umfrage nicht erkennen, in etwas mehr als der Hälfte der teilnehmenden Kliniken werden Kinder postoperativ in einer Kinderklinik untergebracht, eine eigenständige Versorgung von HNO-Kindern separiert von Erwachsenen ist unüblich [1].

Die heterogene postoperative Versorgung von Kindern in Deutschland schließt eine hohe Versorgungsqualität nicht aus, sie spiegelt jedoch die unterschiedliche Infrastruktur sowie die regionale demografische Entwicklung und die daraus erwachsene Aufbau- und Ablauforganisation wider. Die Ambulantisierung HNO-ärztlicher Eingriffe bei Kindern (z. B. Adenotomie, Paukenröhrcheneinlage, Tonsillotomie) hat zu einer deutlichen Minderung HNO-ärztlich-pädiatrischer Bettenkapazität geführt, sodass solitäre HNO-Kinderstationen organisatorisch und ökonomisch problematisch wurden.

Um die Diskussion zur individuellen Konzeptionierung der stationären Betreuung von HNO-Kindern zu erleichtern, schlagen wir vor, eine Klassifikation der Eingriffe anhand der zu operierenden Krankheitsbilder vorzunehmen. Aus der Klassifikation in „Basiseingriff“ und „Komplexeingriff“ ergibt sich dann das Anforderungsprofil an die versorgenden Kliniken bzw. Abteilungen, um die bestmögliche Versorgungsqualität zu erzielen. Für postoperative Notfallsituationen wie Blutungskomplikationen oder eine Verlegung der Atemwege muss ein Notfallplan vorgehalten, transparent gemacht und jährlich/anlassbezogen aktualisiert werden. Der Notfallplan muss interdisziplinär und berufsgruppenübergreifend erstellt und konsentiert werden. Die zeitnahe Verfügbarkeit eines HNO-Arztes sowie die räumliche Nähe und rasche Erreichbarkeit eines Operationssaals/Eingriffsraums mit Anästhesiekapazität sind elementare Kriterien für ein adäquates Notfallmanagement. Prinzipiell muss das betreuende Pflegepersonal im Umgang mit den zugedachten Krankheitsbildern geschult sein. Dieses umfasst die sachgerechte Beobachtung und Fähigkeit, sich anbahnende kritische Situationen zu registrieren, um im Notfall professionell agieren zu können.

Zur Graduierung von pädiatrischen HNO-Prozeduren wird folgende Einteilung vorgeschlagen:

### Basiseingriffe

Die Eingriffsarten gehen über die Inhalte des individuellen Kompetenzerwerbs nach dem Weiterbildungskatalog im Fachgebiet der HNO-Heilkunde und Anästhesiologie nicht hinaus und werden in Hauptabteilungen, Belegabteilungen und Universitätskliniken ausgeführt. Eine HNO-fachärztliche Dauerpräsenz ist nicht erforderlich, ebenso wenig intensivmedizinisches Spezialwissen. Die Unterbringung im Krankenhaus muss sich an den möglichen postoperativen Komplikationen und ihrer schnellstmöglichen Versorgung orientieren. Die pädiatrischen HNO-Patienten sollten prinzipiell von HNO-Ärzten betreut werden, für Besonderheiten ist die Kompetenz von Kinderärzten konsiliarisch hinzuzuziehen. Es ist sinnvoll, die ökonomischen Aspekte unabhängig vom Ort der Unterbringung des Kindes den interdisziplinären Leistungen in der internen Verrechnung anzupassen. Die poststationäre Betreuung erfordert kein HNO-fachärztliches Spezialwissen und ist in der Regel zeitlich limitiert. Beispiel: Tonsillenchirurgie, Paukenröhrchen. Einige dieser Eingriffe werden ambulant durchgeführt. Auch in der ambulanten Durchführung muss HNO-ärztlich und anästhesiologisch eine Kompetenz für pädiatrische Eingriffe/Narkosen vorhanden sein. Ein striktes Qualitätsmanagement ist auch im ambulanten Bereich zu erstellen und vorzuhalten.

Beispiele für Basiseingriffe: Tonsillenchirurgie inklusive Adenotomie, Paukendrainagen, Mittelohrchirurgie – ausgenommen Missbildungen, kleine Fehlbildungschirurgie wie Halszysten/-fisteln oder präaurikuläre Fisteln, Otoplastiken, Lymphknotenexstirpationen, Cochleaimplantationen.

### Komplexeingriffe

Die Eingriffsarten gehen über die Inhalte des individuellen Kompetenzerwerbs nach dem Weiterbildungskatalog hinaus, sind mit postoperativ potenziell lebensbedrohlichen Risiken verbunden und/oder erfordern eine spezielle apparative/personelle Vorhaltung und werden nur in wenigen Hauptabteilungen und Universitätskliniken ausgeführt. Häufig handelt es sich um Eingriffe, auf die sich wenige Operateure in Deutschland spezialisiert haben. Die anästhesiologische/intensivmedizinische/pädiatrische Abteilung muss im Umgang mit den behandelten Krankheitsbildern erfahren sein und eine ärztliche Dauerpräsenz in der Form vorhalten, dass eine interdisziplinäre Notfallversorgung zu jedem Zeitpunkt möglich ist. In Abhängigkeit vom Grad der Komplexität ist oft eine Anbindung der Kinder an ein interdisziplinäres Zentrum mit allen Formen der ärztlichen, nichtärztlichen und apparativen Maximalversorgung angezeigt. Die postoperative Betreuung ist von der operierenden HNO-Klinik federführend zu organisieren und wird von der dominierenden medizinischen Problematik bestimmt. Die poststationäre Betreuung erfordert HNO-fachärztliches Spezialwissen und in der Regel längerfristige HNO-fachärztliche Kontrolluntersuchungen. In besonders komplexen Fällen, beispielsweise in der Sarkomchirurgie, ist eine ärztliche Dauerpräsenz der beteiligten Abteilungen und die Unterbringung auf Spezialstationen mit geeigneter räumlicher/apparativer Versorgung sowie besonders geschultem ärztlichem und nichtärztlichem Personal erforderlich.

Beispiele für Komplexeingriffe: alle Operationen mit der Notwendigkeit einer postoperativen Intensivüberwachung, syndromale PatientInnen, Chirurgie von Atemwegsstenosen, Sarkomtherapie, Missbildungschirurgie, Entfernung von Atemwegsfremdkörpern, onkologische Operationen inklusive rekonstruktiver Chirurgie, Schädelbasischirurgie inklusive Angiofibromentfernungen.

Die Infrastruktur der HNO-Abteilungen in deutschen Kliniken ist im Hinblick auf eine interdisziplinäre Ausrichtung durchaus unterschiedlich und hängt stark von standortindividuellen Parametern und Kompetenzen ab. Die Zuweisung von Kindern in entsprechende Zentren sollte sich stets nach den für die Versorgung vorzuhaltenden interdisziplinären Fachrichtungen und der spezifischen Kompetenz der darin integrierten HNO-Abteilung richten. Eine bestmögliche Versorgungsqualität ist dann zu erreichen, wenn die Zuweisung von Kindern an ein Zentrum diese oben genannte Kompetenzgraduierung berücksichtigt. Zu diskutieren ist außerdem eine nationale Vernetzung von spezialisierten HNO-Kinderabteilungen nach dem Vorbild der durch die Europäische Union geförderten European Reference Networks, da gerade die Diagnostik und das Management seltener Krankheitsbilder nicht von allen HNO-Kliniken abgebildet werden können. Hierzu ist es erforderlich, dass entsprechende Abteilungs- und Zentrumskompetenzen klar kommuniziert und dadurch einfach erkennbar werden.

Die Diskussion mit den Krankenhausträgern und den interdisziplinären Partnern über die optimale postoperative Betreuung von Kindern nach einem HNO-chirurgischen Eingriff wird teilweise durchaus emotional geführt. Die Beweggründe für die Etablierung entsprechender Konzepte sind zwar standortspezifisch unterschiedlich, allerdings sollte die Festlegung von Überwachungsstrategien ausschließlich patientenzentriert stattfinden. Vorliegende Stellungnahme kann diese Diskussion argumentativ stützen und eine von der Grundstruktur her einheitliche Herangehensweise an eine Konzeptionierung unter Berücksichtigung der sehr heterogenen Versorgungssituation in Deutschland ermöglichen.
